# Strategies and approaches to vector control in nine malaria-eliminating countries: a cross-case study analysis

**DOI:** 10.1186/s12936-015-1054-z

**Published:** 2016-01-04

**Authors:** Cara Smith Gueye, Gretchen Newby, Roland D. Gosling, Maxine A. Whittaker, Daniel Chandramohan, Laurence Slutsker, Marcel Tanner

**Affiliations:** Malaria Elimination Initiative, Global Health Group, University of California, San Francisco, 550 16th Street, 3rd Floor, San Francisco, CA USA; Centers for Disease Control and Prevention, 1600 Clifton Road, Atlanta, GA USA; London School of Tropical Medicine and Hygiene, Keppel Street, London, WC1E 7HT UK; The University of Queensland School of Public Health, Herston, QLD Australia; Swiss Tropical and Public Health Institute, Socinstrasse 57, 4051 Basel, Switzerland; University of Basel, Basel, Switzerland

**Keywords:** Malaria, Elimination, Eliminating, Control, Vector, Vector control, Entomology, Surveillance, Indoor residual spraying, Long-lasting insecticidal nets

## Abstract

**Background:**

There has been progress towards malaria elimination in the last decade. In response, WHO launched the Global Technical Strategy (GTS), in which vector surveillance and control play important roles. Country experiences in the Eliminating Malaria Case Study Series were reviewed to identify success factors on the road to elimination using a cross-case study analytic approach.

**Methods:**

Reports were included in the analysis if final English language draft reports or publications were available at the time of analysis (Bhutan, Cape Verde, Malaysia, Mauritius, Namibia, Philippines, Sri Lanka, Turkey, Turkmenistan). A conceptual framework for vector control in malaria elimination was developed, reviewed, formatted as a matrix, and case study data was extracted and entered into the matrix. A workshop was convened during which participants conducted reviews of the case studies and matrices and arrived at a consensus on the evidence and lessons. The framework was revised and a second round of data extraction, synthesis and summary of the case study reports was conducted.

**Results:**

Countries implemented a range of vector control interventions. Most countries aligned with integrated vector management, however its impact was not well articulated. All programmes conducted entomological surveillance, but the response (i.e., stratification and targeting of interventions, outbreak forecasting and strategy) was limited or not described. Indoor residual spraying (IRS) was commonly used by countries. There were several examples of severe reductions or halting of IRS coverage and subsequent resurgence of malaria. Funding and operational constraints and poor implementation had roles. Bed nets were commonly used by most programmes; coverage and effectiveness were either not measured or not articulated. Larval control was an important intervention for several countries, preventing re-introduction, however coverage and impact on incidence were not described. Across all interventions, coverage indicators were incomparable, and the rationale for which tools were used and which were not used appeared to be a function of the availability of funding, operational issues and cost instead of evidence of effectiveness to reduce incidence.

**Conclusions:**

More work is required to fill gaps in programme guidance, clarify the best methods for choosing and targeting vector control interventions, and support to measure cost, cost-effectiveness and cost-benefit of vector surveillance and control interventions.

## Background

Tremendous progress has been made over the last decade in reducing morbidity and mortality from malaria. At present, 55 countries are on track for or have already achieved a 75 % reduction in morbidity from 2000 to 2015 [1]. This progress has prompted a review of the current global malaria strategy and goals, set forth in the *Global Technical Strategy for Malaria 2016*–*2030* (GTS) by the Global Malaria Programme of the World Health Organization (WHO) and its implementation and action framework, *Action and Investment to Defeat Malaria* (AIM) by Roll Back Malaria (RBM). GTS was approved by the World Health Assembly in May 2015 and AIM by the RBM Advisory Board in the same month [2, 3]. Out of the three pillars laid out in the GTS to ensure continued progress towards and achievement of malaria elimination, two emphasize the role of entomological surveillance and vector control response.

Vector control encompasses the measures that are directed against a vector of disease, intended to limit its ability to transmit the disease by protecting areas that are known to be receptive to transmission [4]. Receptivity to malaria depends on the vectorial capacity of local vector populations, as in not just the presence of the vector but its population size, human biting habits and longevity in relation to the period of sporogony. Each of these parameters is strongly influenced by the climate, local ecology and behaviour of both humans and vectors. In an elimination phase, the objective of vector control is the reduction of the vectorial capacity of the local vector populations below the critical threshold needed to maintain transmission [5].

**Fig. 1:**
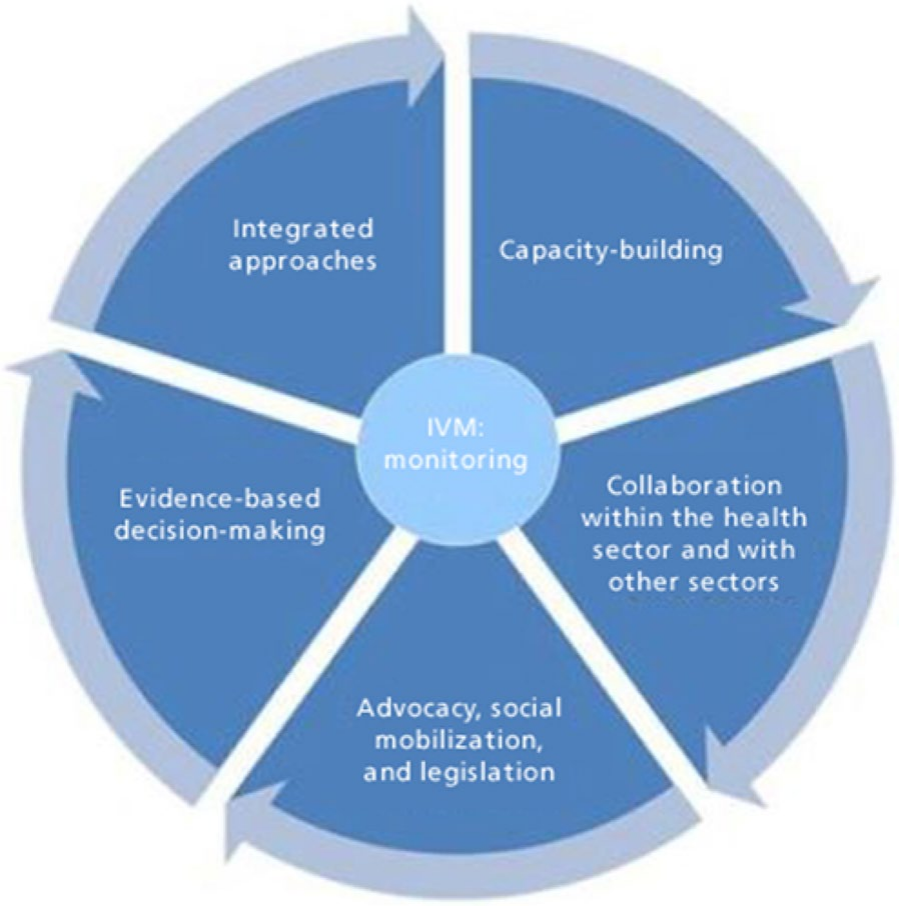
IVM framework and distinguishing characteristics. Source: Beier et al. [7]

The GTS outlines the need for high-quality implementation of core vector control tools of indoor residual spraying (IRS) and long-lasting insecticide-treated bed nets (LLINs), as well as the role of larval source management as a supplementary tool. Integrated vector management (IVM) should be the overarching vector control strategy for all countries, and includes the components described in Fig. 1 [6, 7].

Routine entomological surveillance (e.g., vector mapping and bionomics) and insecticide resistance monitoring data should be combined with epidemiological data to identify new vectors or shifts in vector composition, understand receptivity in a country setting, inform choice of vector control interventions, coverage, timing, and to evaluate the quality and impact of interventions. When malaria burden is reduced to low levels, a shift from universal to targeted vector control activities is needed for those programmes that are ready for this transition. Plans must be in place for the management of insecticide resistance, operational research to develop and validate new tools, as well as strategies to improve upon microstratification and delivery of interventions.

As vector control is an important component in the overall strategy to control and ultimately eliminate malaria, there may be factors in its implementation that influence the likelihood of attaining malaria elimination. Vector control intervention choice and how it matches the context of vector habitat and behaviours, targeting and coverage of at-risk populations, and evaluation and modification of programme interventions may influence the success or failure of malaria elimination programmes. The Eliminating Malaria Case Study Series by the WHO Global Malaria Programme and University of California, San Francisco (UCSF) Global Health Group provides detailed examples of national malaria programmes that are currently eliminating or have eliminated malaria, offering an opportunity to review synthetically key components of these programmes. In this paper a review of vector control activities across nine countries was undertaken to identify success factors along the road to elimination using a cross-case study, analytic approach. The analysis focuses on vector control tools, approaches, coverage and, when information was available, impact in elimination settings.

## Methods

This cross-case study review included nine case studies from the Eliminating Malaria Case Study Series, produced through a collaboration between the WHO Global Malaria Programme and UCSF Global Health Group. Each case study details the programme strategies and interventions from the early 1900s to the current period, with epidemiological and intervention data coverage and an analysis of the main factors behind their successful handling of outbreaks or epidemics and programmatic challenges. Countries were selected for the case study series if they: (a) demonstrated successful transition towards or achievement of elimination; (b) committed to the case study research and analysis process; and, (c) were able to provide access to sufficient data. Countries were also chosen to represent a range in malaria epidemiology, stage of elimination (from low endemic control to prevention of re-introduction), geography (island *vs* continental), and strength of their health system. Countries selected were Bhutan, Cape Verde, Malaysia, Mauritius, Namibia, Philippines, La Reunion, Sri Lanka, Tunisia, Turkey, and Turkmenistan [8–16]. Table 1 shows the different stages and goals of the nine countries that were included in this review. Prevention of re-introduction (POR) countries were those considered to have reached zero locally acquired cases and are actively preventing re-introduction of malaria [4].

**Table 1:** Elimination history and goals of the nine case study countries

Country	Elimination status	Elimination history
Bhutan	Eliminating	Goal of zero transmission nationally by 2018; national malaria elimination certification by 2020
Cape Verde	Eliminating	Achieved zero cases 1968–72 but epidemic occurred during 1977–79. Second elimination attempt 1983–85, however epidemic occurred during 1987–88. Goal of national elimination by 2020
Malaysia	Eliminating	Goal of national elimination by 2020: elimination in West Malaysia by 2015 and elimination in Sabah and Sarawak by 2020
Mauritius	Prevention of re-introduction	First eliminated in 1969 and received WHO certification in 1973. Resurgence in 1975. Second elimination achieved by 1998
Namibia	Eliminating	Goal of national elimination by 2020
Phili-ppines	Eliminating	Strategy of progressive sub-national elimination with national elimination (all provinces) by 2025 (recently updated to 2030)
Sri Lanka	Eliminating	Near elimination in 1963, then an epidemic from 1967 to 68. Zero local cases reported since November 2012; will seek WHO certification by end of 2015
Turkey	Prevention of re-introduction	Most of the country in consolidation phase in 1974, followed by epidemics in 1977 and 1993–1996. Last indigenous cases reported in 2012 during outbreak
Turkmen-istan	Prevention of re-introduction	First eliminated in 1961. In most recent attempt, the last indigenous case occurred in 2004. Received WHO certification in 2010

Case studies were included in the cross-case analysis if they were part of the WHO Global Malaria Programme/UCSF Global Health Group case study report series, all of which used the same type of quantitative and qualitative approaches and methods. Reports or publications that were in final English language draft at the time of analysis (November 2014) were included. Case studies included in this cross-case analysis are Bhutan, Cape Verde, Malaysia, Mauritius, Namibia, Philippines, Sri Lanka, Turkey, and Turkmenistan. Case studies from La Reunion and Tunisia were not included in the cross-case study review because the report from La Reunion was not finalized nor translated into English at the time of analysis, and a draft of Tunisia was not yet available by the time the analysis was underway.

A conceptual framework for vector control in malaria elimination was developed to provide structure for the cross-case analysis. To develop this framework, a document review was conducted of malaria elimination vector control guidelines, reports, consultations, and manuals to identify historical and current policy and research on vector control strategies, entomological surveillance, operational research, and costs. Search terms included ‘vector control’ and ‘malaria elimination’ or ‘malaria’; or ‘indoor residual spraying’, ‘insecticide-treated nets’, ‘long-lasting insecticide treated nets’, ‘entomology’, ‘entomological surveillance’, ‘larval control’, and ‘larval source management’ in the following search engines and databases: The Cochrane Library, PubMed, Google Scholar, and WHOSIS. Using this literature, a conceptual framework of vector control strategies and interventions was developed based on the topic areas of vector species and behaviour, approach to vector control, tools and coverage, combination interventions, stratification, outbreak response, implementing organizations, and cost of activities. The framework was reviewed by malaria elimination and vector control experts and formatted in Excel as a matrix. A first round of data extraction from the case study reports occurred as a result of a thorough review of the nine reports by two researchers (CSG, GN). CSG and GN then extracted challenges and weaknesses of the vector control programme for each case study and reviewed each other’s summaries. This analysis focused on the vector control strategies and tools used after the Global Malaria Eradication Programme (GMEP, 1955-1970), in order to reflect current tools (e.g., LLINs) and research.

Once the matrices with data and summaries were assembled, a two-day workshop of malaria elimination researchers and experts was convened to review the case studies, matrix summaries and findings to ensure that the data captured in the matrix were comprehensive and to debate the different learning across the country experiences. Workshop participants revisited the principles of vector control (aims, objectives, what implemented, how implemented, by whom) and identified examples from each case study report for each of the elements of the framework, arriving at a consensus on the evidence and lessons learned from the case study series. A second round of data extraction and summary was undertaken to ensure that data was extracted for each portion of the framework. The results of the cross-case analysis were then compared with the strategies laid out in the GTS.

## Results

The review of case studies showed that all countries implemented a range of vector control interventions, whether they had eliminated (Mauritius, Turkey, Turkmenistan) or were moving towards elimination (Bhutan, Cape Verde, Malaysia, Namibia, Philippines, Sri Lanka). The types of intervention used were likely determined by many factors, including operational constraints, cost, vector density and behaviour, insecticide resistance levels and epidemiological trends, among others. The vector control tools used by each country can be found in Table 2.

**Table 2:** Vector control intervention mix across the nine case study countries

	GTS tool	Bhutan	Cape Verde	Malaysia	Mauritius	Nambia	Philippines	Sri Lanka	Turkey	Turkmenistan
Primary vectors		*An. pseudowillmori* *An. culicifacies*	*An. arabiensis (An gambiae complex)*	West Malaysia: *An. maculatus* Sabah: *An. balabacensis* *Sarawak: An. latens, An. donaldi*	*An. gambiae s.l.* (identified as *An. arabiensis* in 1975)	*An. arabiensis*	*An. flavirostris* *An. maculatus*	*An. culicifacies (species E)*	*An. sacharovi* *An. superpictus*	*An. pulcherrimus* *An. superpictus*
IVM	X	2		2				2		2
Entom. Surv.	X	2	2	1	1 C	2	2	1	2	1
Response to entom.surv.	X			2	2		2			
IRS	X	1	2	2	2	1	2	1	2	2
Thermal fogging					2			2	2	
LLIN/ITN	X	1		2^a^		1	2	1	2	2^b^
Larv. fish	X		2				2	2	1	C
Larviciding	X	2	2	2	1	2	2	2	1	
Env. mgt.			2	2	2		2	2	2	2
Personal protection							2			2

*GTS* Global Technical Strategy

*1* Primary vector control intervention during most recent elimination strategy

*2* A vector control intervention implemented during recent elimination programme, but not considered primary

*C* Strategy used during consolidation phase (after having achieved elimination)

^a^ITN only

^b^Locally produced bed nets

The IVM strategy document was disseminated by WHO in 2004 [17]. Most countries that were eliminating or had eliminated had strategies in place that used components of IVM, in particular the combination of interventions. IRS, insecticide-treated nets (ITNs) and LLINs were used commonly by most programmes to collectively increase population coverage, along with larval control. Some countries supplemented these interventions with environmental management, personal protection and insecticide fogging. Implementation most typically occurred at the district level, with guidance and strategy development provided at the national level. Some reports showed outsourcing of vector control activities to community volunteers or the private sector. There was little explicit description of the other four components of IVM, such as collaboration in health and with other sectors; advocacy, social mobilization and legislation; capacity building; nor development and use of evidence-based decision-making.

The rationale for which tools were used and which were not used was not well-articulated in the case studies. Moreover, there did not appear to be a clear linkage between entomological surveillance data, including insecticide resistance data, and parasitological data, nor was there evidence that either types of data informed intervention choice. Instead, the availability of funding and cost of interventions appeared to have played an important role in decision making for vector control interventions. The coverage and targeting of interventions was also poorly reported in the case studies. Some case studies included detailed stratification strategies, but not all. Even for those with a stratification strategy, most case studies did not consistently report on intervention coverage, and the ways in which coverage was described varied enormously, making comparisons across time periods and countries difficult. There was little evidence of reported quality assessment of interventions.

Measurement or evidence of impact of vector control interventions was scant or practically absent. Many case studies indicated that activities were effective in reducing receptivity in risk areas, but did not provide evidence or indicators, instead using anecdotal evidence that was likely based on programme experience.

In the analysis, the targeting, coverage and impact of all vector control measures were compared across the case study countries and similarities and differences highlighted. The results are described below for each vector control approach and tool.

### Integrated vector management

IVM was adopted by four of nine programmes in the cross-case study analysis, but the meaning and utility of IVM varied across case studies (Table 3). The strategy of IVM was introduced in 2004 by WHO to increase cost effectiveness of vector control and to reduce the spread of drug and insecticide resistance [17]. The strategy focused on using a combination of interventions to attack the vector at different stages of its life cycle. It also requires decisions on which tools to use to be made based on evidence and that the type of vector control deployed will change as one approaches elimination and post-elimination (Fig. 1). For some countries (e.g., Turkmenistan) it was used as a way to combine vector control interventions. In other countries it ensured intersectoral collaboration, community engagement and integration of services, such as entomological surveillance, with other diseases (e.g., dengue). In Sri Lanka, IVM combined all of these elements, and engaged other sectors and communities in developing vector control strategies. It also ensured the use of a mix of interventions, as well as insecticide rotation for IRS, in which different types of insecticides were used in bordering districts with rotation of insecticides across districts over time, in order to lessen the risk of the development of insecticide resistance.

**Table 3:** Integrated vector management adoption and definition

	Bhutan	Cape Verde	Malaysia	Mauritius	Namibia	Philippines	Sri Lanka	Turkey	Turkmenistan
Implementa-tion of IVM and timeline	X (National Five-Year Plan 2008–2013)		X (2011)				X (Mid 1990s)		X (1998)
Components of IVM implemented									
Intersectoral collaboration			X				X		
Community engagement	X						X		
Insecticide rotation							X		
Combination of vector control interventions	X						X		X

The impact of IVM was not articulated in the reports, except for Sri Lanka, where the use of the approach in agricultural areas was thought to have contributed to a reduction in malaria incidence. Further research would be valuable to understand the impact of implementation of IVM as a broad strategy on reducing malaria transmission.

### Entomological surveillance

Most countries in the case study series began conducting entomological surveillance during the GMEP. Entomological surveillance is typically comprised of monitoring of larval habitats, surveying for adult mosquitoes, conducting insecticide susceptibility tests, and assessing changes in environmental parameters [4], with the objectives of identifying the level of change in receptivity, and of designing and monitoring effectiveness of programme vector control strategy and interventions. The case studies did not outline specific activities that were maintained in the current elimination periods, instead only providing details and time frame when a new effort or initiative was undertaken. Even for countries that had more consistent entomological surveillance, the response component was not articulated in the case studies; it appears that, for most countries, entomological surveillance data were not analysed and used for outbreak forecasting or programme strategy, including better targeting of vector control interventions.

There was variation in the quality and consistency of entomological surveillance across the case studies. Countries that have reached elimination generally had a more detailed description of their surveillance programmes. For example, in the years leading up to elimination in Turkmenistan (2004–10), the programme maintained ‘passports’ for each water body, and district officials systematically updated a database on vector bionomics and densities. Entomological officers were recruited to serve on epidemic response mobile teams. In Turkey, surveillance included mapping of larval habitats in addition to data collection in sentinel sites. The continuation of this type of surveillance through the years of POR and post-elimination certification was only described in detail in the Mauritius report, where the programme maintained weekly surveillance of breeding areas since elimination in 2008.

The Malaysia and Sri Lanka case studies likewise described strong entomological surveillance programmes. In both countries, consistent entomological surveillance was one of several approaches credited by the malaria programmes for the national progress in reducing incidence, as it was used to guide planning of vector control. Malaysia’s diversity of vectors was a reason for continual monitoring, and district-level surveillance tracked larval habitats (conducted by district entomologists and assistant environmental health officers). Mapping with GPS units captured housing locations and larval habitats. Sri Lanka’s national and district health offices conducted entomological surveillance on a monthly basis. In later years, Sri Lanka had a large increase in funding to support entomological surveillance (from a Global Fund grant), which was conducted by a private sector organization in some areas. In Bhutan, surveillance was conducted monthly.

In other countries, entomological surveillance was more limited, such as in Cape Verde, where there was not a consistent programme of monitoring. Surveillance in the Philippines was limited to semi-annual or annual monitoring in the sporadic and malaria-prone transmission provinces.

In all case study countries, data collected during surveillance were not consistently used by programmes. Most case studies did not describe the use of entomological surveillance data to assess impact of interventions or to inform programme strategy. For example, because Turkey did not conduct entomological evaluations pre- and post-epidemic (after 1993), the programme was unable to assess effectiveness of the response interventions. There are some examples of programmes using their entomological data to guide decision-making. In the Philippines, surveillance data were reviewed during sub-national, provincial elimination certification, a process that was formalized in 2011. In addition, prior to the national programme’s devolution, all new strategies were tested through field research and entomological and parasitological surveys before becoming policy, such as the shift from IRS alone to combined IRS with ITNs. Bioassay and susceptibility test results guided changes in insecticide usage. In Malaysia and Mauritius, maps of larval habitats were used to target vector control interventions. Also in Malaysia, research was undertaken by district and state officers to measure effectiveness of management of the larval stage of the vector in reducing receptivity, although the outcomes of this research were not described in the report.

As entomological surveillance data should be the basis for all response interventions and programme strategies, consistent and high-quality data are needed. Further action is required to ensure that entomological surveillance is a priority for elimination programmes and that data are analysed and inform robust response, including forecasting, targeting and programme strategy.

### Indoor residual spraying

Each of the nine programmes employed IRS, and most countries continued IRS after its introduction during the GMEP era because IRS historically was found to be effective in reducing receptivity. IRS targeting strategies varied across the countries, but generally by the 1990s most countries had transitioned to focal IRS instead of universal coverage, or blanket spray, operations. This transition may have been in response to the introduction of the WHO Global Strategy for Malaria Control [18]. As all countries (both eliminating and POR) approached elimination, their programmes transitioned to targeting IRS for active foci or active transmission areas.

In the case studies there were several instances of premature reduction of coverage or disbanding of IRS, some of which were linked to subsequent resurgences of malaria (e.g., Cape Verde, Sri Lanka, Turkey). The reported reasons for reducing IRS operations varied, but the trend was that scale-down occurred when countries were very close to eliminating malaria or were firmly in the POR stage. In Sri Lanka, IRS was halted in eliminated areas, which is thought to have contributed to the epidemic of 1957. In more recent times, Sri Lanka has shown a decline in IRS coverage as it moved from full coverage of risk areas to focal IRS (conducted in areas with malaria cases) and outbreak response, moving from 23 % coverage of total population in 2005 to 6 % in 2010. Even without continued IRS coverage, however, to date Sri Lanka has been able to maintain low caseload and has not experienced a resurgence, perhaps related to the continued distribution of LLINs and use of larval control in addition to a strong surveillance system. In Cape Verde, in contrast, twice in recent history, foci on Santiago Island were re-activated within 3 years after relaxation of aggressive, bi-annual IRS operations. IRS was not replaced by another vector control intervention; larval control (temephos and larvivorous fish) was used after the 1960s in Cape Verde, but there is no evidence in the case study that it was scaled up when IRS declined, and coverage data were not available. Cape Verde has since continued its small-scale IRS operations, mainly outbreak response activities that covered about 5–10 % of Santiago Island.

Turkey scaled down IRS to residual foci only when it did not achieve elimination during the GMEP, and in the 1970s and 1990s fell short of coverage of active foci that was achieved in 1961 (86–88 %) and 1968 (nearly 100 %). In both the 1970s and 1990s, reductions in IRS coverage were linked to the availability of funding; the malaria service was under pressure to reduce expenses when it did not reach elimination. Other challenges included operational constraints, lower quality of implementation, a high rate of refusals in the target population, and insufficient and inexperienced staff. IRS was not replaced by another method of vector control at that time, although larviciding had been used as a complementary measure since the late 1950s. In its latest strategy, the country reserved IRS for areas with residual or active transmission. Likewise, Mauritius did not have enough funding to conduct IRS island-wide during its second elimination attempt, so it was restricted to areas with ongoing transmission. Mauritius used a combination of interventions (IRS, fogging, larval control, and entomological surveillance) for areas with transmission that reported more than three cases. Areas with fewer than three cases did not receive IRS. Coverage was described as 65–80 % of foci in 1986, although it was not clear in the case study if this was considered sufficient. In recent years, Mauritius used IRS to prevent establishment of transmission within a residence of a confirmed case, of which all are imported.

Some countries, particularly those in the early stages of elimination, indicated that operational constraints, instead of a stratification strategy, led to the scale-down of IRS. Worker shortages and an inability to mobilize spray teams, inadequate training, and low morale were all factors described in the case studies. In the 1990s, the Philippines reduced IRS coverage to 20 % of targeted areas as a result of operational disruptions during the process of programme decentralization. Even when an increase in funding boosted coverage to two spray cycles per year with 76 % of target achieved, quality was considered poor due to delays, lack of training, and an insufficient number of spraymen. In part because of the operational challenges and in part due to Global Fund influence, the country focused instead on LLIN distribution. In 2011, ITN and LLIN coverage in the 40 target provinces was 73 % of the total target population. In Namibia, rainy conditions, poor roads and worker shortages have prevented completion of IRS activities. IRS national coverage of at-risk populations ranged from 16 to 41 % from 2001 to 2011, and the country revised its goal to a target of 95 % coverage in areas of moderate endemicity and 100 % focal coverage in low-endemic regions, prioritizing the highest burden villages in the event that the spray season was cut short due to staffing or logistics problems. In Bhutan, political instability in the southern region in the early 1990s led to difficulties in completing IRS spray campaigns and by 1994 cases were increasing. IRS was halted in 1998 when the programme switched to ITNs as a primary vector control measure. Focal IRS was re-instated in 2004 and by 2012 the Bhutan programme reported achieving 100 % coverage of its target population (14 % of the population at risk).

Some countries appear to have maintained a consistent level of coverage. Turkmenistan employed IRS as an outbreak response measure, covering 91–100 % of targeted areas during the 1998–2000 period. The programme did not conduct IRS from 2005 because there were no malaria infections to ‘trigger’ the focal IRS response. The case study on Malaysia did not report any decline in IRS activities, but it was challenging to understand the coverage because it was measured as the number of households sprayed of those targeted, and not by proportion of risk population protected.

Some programmes relied on communities or volunteers for IRS campaigns, such as in the Philippines. Bhutan also trained community volunteers to conduct IRS, however the quality and coverage declined so volunteer teams were disbanded. In some private sector plantations in Sabah State (Borneo) of Malaysia, IRS was implemented (and paid for) by the plantations, with oversight by the Sabah Malaria Control Programme.

Effectiveness of IRS to reduce receptivity was assumed in the reports, evidenced by declines in malaria incidence in the 1950s and 1960s that were linked with increases in IRS coverage. But the picture became more complicated in recent years, as multiple interventions were employed at the same time. This was the case in Malaysia, where IRS with ITN distribution (ITN distributed began in 1995) was credited for a decrease in annual parasite index (API), the number of reported cases per 1000 population per year, from 3.0 (1995) to 0.5 (2000), in addition to the benefits of replacing DDT with pyrethroids in 1998. Turkey and Mauritius also attributed malaria case declines to IRS activities along with active surveillance measures.

Most case study reports did not contain adequate information on recent insecticide resistance monitoring activities or description of evidence of resistance. Malaysia and the Philippines described the sentinel sites for monitoring insecticide resistance. Malaysia, Namibia, and the Philippines reported conducting bioassay and susceptibility tests on insecticides. In the Philippines, Laguna Province shifted insecticides reportedly due to a drop in effectiveness after 10 years, and more recently there was pyrethroid resistance possibly detected in Isabela Province. Sri Lanka implemented insecticide rotation in 1998, part of IVM, in order to prolong the life and utility of the insecticides and optimize vector control.

Given the experience of several countries that halted or scaled down IRS and suffered serious epidemics and resurgences of malaria, further research is needed on the transmission dynamics in various types of contexts, and the alternative methods, such as larval source management, that can be put into place to avoid resurgence. Information should also be shared on the monitoring for insecticide resistance and the programmatic response to the data collected. For some countries, typically higher endemic areas, logistical issues or decreases in funding have led to poor quality implementation or disruption of IRS. Less resource intensive, sustainable methods for vector control must be explored for some countries.

### Space spray

Outdoor space spray with insecticide was reported in the case studies of three programmes: Mauritius, Sri Lanka and Turkey.

Mauritius used space spray as an epidemic response measure starting in 1975, but by 1981 it was discontinued. Implementation was viewed as costly and ineffective because it was conducted in the morning when the temperature was too warm. The thermal clines made the insecticide rise and in addition the mosquitos were not flying at that time. It was re-instated in 1982 as a response to the outdoor-biting behaviour of *Anopheles gambiae s.l.*, this time conducted in the evening. At that time, coverage was limited to the Port Louis areas in response to outbreaks only. In Sri Lanka, space spray has been used during festivals and other large gatherings, but coverage and effectiveness was not articulated in the case study. Turkey conducted space spray as an outbreak containment strategy. While the report indicated that epidemics were controlled through a combination of interventions that included space spray, there are no data on the effectiveness of space spray alone. More research specifically on the impact on malaria transmission of space spray in countries that use it would help in developing an evidence base.

### Long-lasting insecticidal nets/insecticide-treated nets

Most malaria programmes in the case study series employed ITNs, followed by LLINs as they became available, as a supplementary vector control measure to IRS. However, the countries in POR (Mauritius, Turkey, Turkmenistan) never used ITNs or LLINs, as they had achieved elimination before they were available. One exception is Turkmenistan, where locally made bed nets were in use since the 1930s and were reportedly widely used (coverage rates not given) in the 2004–2010 elimination campaign.

Of the six eliminating countries, Cape Verde never employed LLINs or ITNs, although information on the reasons behind this was not reported. ITNs/LLINs became a primary vector control tool in the Philippines and Namibia, and replaced IRS for 6 years in Bhutan (1998–2004), until cases doubled from 1998 to 1999, sparking a programme review and the introduction of several activities, including focal IRS to supplement ITNs. The programme had struggled to re-treat ITNs in a timely manner, which may have contributed to the increase in cases. Malaysia never switched from ITNs to LLINs because the programme believed that ITNs were sufficient. Malaysia also did not have external funding, such as a Global Fund grant, which may have contributed to the decision to continue ITN use. LLINs have been used to protect populations living or working in hard-to-reach or remote areas, such as parts of Bhutan and in the former conflict zone of Sri Lanka. NGOs in Sri Lanka that were familiar with the conflict-affected communities in the east and north distributed LLINs.

Similar to reporting on IRS coverage, comparison of coverage and its definition for ITNs/LLINs across case studies was challenging. Countries used different estimates, most based on net ownership rather than any measure of use, including the number of nets distributed as a proportion of the national total population or national population at risk. Only the Philippines case study report detailed the assumptions behind the LLIN coverage indicator. In the Philippines, coverage was defined as two people having an LLIN for an assumed net lifespan of 3 years. In Sabah, one of the most endemic areas of Malaysia, 55 % of the high-risk areas were considered covered by ITNs in 2009. The distribution of ITNs then increased, from 56,000 in 2009 to nearly 80,000 in 2011, while continuing re-treatment of older ITNs. In Sri Lanka, LLINs were introduced in 2004 and by 2005 15 % of the population at risk, approximately 440,000 persons, was considered to be covered (protected) by a LLIN, climbing to 35 % by 2010. It was believed that the combination of IRS and LLINs in the country helped to lower receptivity. The Philippines programme first distributed ITNs in 1990, then LLINs were introduced in 2005, and by 2011, ITN and LLIN coverage in the 40 provinces that received funding from the Global Fund was 73 % of the target. In Namibia, ITNs were first distributed in 1993 and then replaced by LLINs in the mid-2000s. By 2005, coverage ranged from 5 to 10 % of the population at risk, increasing to 50 % in 2009 and 2010, but dropped down to 30 % in 2011. Mass distribution of nearly 500,000 LLINs in the northern regions was conducted in 2013.

Other alternatives have also been tested. The Philippines experimented with hammock-type LLINs for their military but they found the available design to be too difficult to climb out of so they were not scaled up. Hammock LLINs were found to be an effective tool for preventing malaria in forested areas of Cambodia, but this may be related to cultural factors, as villagers and forest workers in the area were used to using hammocks in the early evening hours [19]. In Sri Lanka, efficacy of insecticide-treated curtains was studied in the late 1990s but no scale up was reported.

ITNs/LLINs have been a core vector control tool for many countries, in particular for populations that are harder to reach with IRS. However, coverage estimates are difficult to compare across countries, and actual use has been difficult to estimate, thus it has been difficult to estimate the impact of ITNs/LLINs. Routine monitoring of coverage and impact of LLINs must be enhanced to better estimate their programmatic impact, especially on a more regular basis, to support locally relevant use of the nets.

### Larval control

Larval control is defined as the use of substances that kill or inhibit the development of mosquito larvae or the introduction of fish or invertebrates that feed on larvae [20], and has been employed by all countries in the analysis. Larval control can include either larvivorous fish or larviciding (which includes both chemical and biological agents in water bodies to kill mosquito larvae).

Most countries started using larval control in the early years of their control programmes (1930s or 1940s) or during the GMEP campaign. Several of the case studies highlighted larval control as a strategy for outbreak or epidemic response (e.g., Bhutan, Malaysia, Mauritius, Turkey, Turkmenistan). In some countries larval control was used as a supplement to IRS, to cover areas that had low or phased-out IRS coverage (e.g., Cape Verde, Mauritius, Sri Lanka), or when zero cases had been reached and IRS was discontinued (Turkey, Turkmenistan). Coverage was typically measured by the number of persons estimated to be protected by this method but this was not detailed in most of the case study reports. When coverage was reported, it was measured in a variety of ways.

In the countries that have eliminated malaria (Mauritius, Turkey, Turkmenistan), larval control has been a continuous and important vector control method and is part of their POR strategic plans. In Mauritius, use of larvivorous fish was perceived to be useful when implemented in proximity to the airport (to lower receptivity in an area that may have imported cases) as well as in deeper rooftop pools and irrigation ponds where vectors were breeding. For the eliminating countries, there were differences in when and why larval control was used. In Malaysia, for example, it was used in low-risk areas throughout the year to keep receptivity at low levels; in contrast, in Namibia it was used primarily in the dry season, when there were fewer water bodies to treat. Sri Lanka used chemical larviciding in abandoned gem pits and wells. Difficulties in implementing larval control were noted throughout the case studies. In Namibia, perceived risk of poisoning animals impeded its widespread use, as did the cost. Inconsistent use of larval control (Philippines and Namibia), lack of intervention data reported to the central level (Cape Verde), lack of breeding site maps (Mauritius), and lack of entomological surveillance in intervention areas (Mauritius) made it difficult to assess the impact of larval control on reducing receptivity or malaria incidence.

Effectiveness of larval control has been measured in Mauritius and Turkey. However, it was conducted in combination with other interventions (in Mauritius alongside IRS and fogging; in Turkey alongside IRS and environmental management) so it was not possible to identify the impact of larval control alone. Research on larval control undertaken in Sri Lanka showed reductions in vector density in the laboratory and in field sites, such as dams, gem pits, brick-making fields, and cement water tanks [21, 22], but the study did not measure impact on malaria transmission.

Similar to IRS and LLINs, coverage of larval control has been measured in different ways across programmes. Countries measured larval control by coverage of larval habitats, hectares, reservoirs, or by the number of people protected, all of which are challenging to compare or understand the scale, much less the impact of this intervention. In Turkmenistan, 136 larval habitats and labour camps (in the early 2000s) were covered by larval control, and (in 2009) six hectares were treated with oil-based larvicides and 1828 hectares were treated with fish. In Mauritius (1985), nearly 16,000 potential larval habitats were treated with temephos. In Sri Lanka in 2001 approximately one million people were estimated to be protected through the distribution of larvivorous fish, but by 2002 only 40,000 were considered to be protected.

As there are some countries that may rely heavily on larval control in the prevention of re-introduction stages, such as Sri Lanka, more rigorous monitoring, including stronger indicators, and measurement of impact is needed to understand the best settings for its implementation.

### Environmental management

Environmental management activities aim to reduce the size of the immature vector population through habitat modification [20]. Environmental modification activities ranged across the case studies, depending on the *Anopheles* species and their preferred larval habitats: cleaning and drainage projects (Bhutan, some parts of Malaysia, Mauritius), marsh draining (Turkey), cleaning or flushing of stream or irrigation canals (Philippines, Sri Lanka, Turkey), infilling of unused reservoirs (Turkmenistan), intermittent drying of reservoirs (Cape Verde), protection of water tanks (Cape Verde), and filling of unused gem pits (Sri Lanka). Namibia did not list any of these activities.

Environmental management was used as a major intervention for five programmes (Turkey, Turkmenistan, Malaysia, Philippines, Sri Lanka) since the early 1900s. In Malaysia it was mainly used in West Malaysia. It was continued as a supplementary measure to IRS in Turkey and Malaysia, as an outbreak response measure in Turkmenistan, and part of the POR strategy in Mauritius. Coverage was not reported in the case studies.

In Mauritius, the large-scale draining/cleaning projects, in addition to other factors such as improvements to housing structures and urbanization, is credited with decreasing the level of malaria transmission before the initial malaria elimination campaign and helped to sustain lower transmission levels during the rest of the 20th Century. In the Philippines, stream clearing was used as a supplementary vector control measure, but had limited overall impact on case incidence, which may be in part due to its inconsistent use.

Similar to larval control, environmental management has been used by many countries as an ongoing vector control tool, and may become more important in the end stages towards malaria elimination. However, as with larval control, methods to monitor its impact on transmission need to be improved.

### Personal protection

Four of the case studies reported having a strategy that included use of personal protection approaches, such as promotion of protective clothing, or insecticide-treated products and some without a strong evidence base, such as ingesting traditional herbal medicines. For example, in the Philippines, use of personal protective measures during evening activities was a recommended strategy, but the specific activities were not described. Namibia promoted awareness in the community of wearing protective clothing, and in one region the population traditionally used herbs as personal protection.

Personal protection methods may become more important in settings where outdoor-biting anophelines play or will begin to play a larger role in transmission, owing partly to vector replacement dynamics. Additional evidence is needed on the effectiveness of these tools on transmission reduction at the community level.

### Economic development and development projects

Economic development was noted as a main contributor to declining receptivity across many countries as it catalyzed changes that impeded the breeding, feeding or resting behaviour of major malaria vectors. Economic development may have led to changes at individual household level (e.g., housing materials) or larger community level (e.g., large-scale construction projects, urbanization, increased access to medical care and services). Improvements in housing made indoor feeding more difficult, as anophelines were less able to enter and exit homes pre- and post-feeding. These improvements, including use of air conditioning by about 50 % of households and villages, were likely contributors to a reduction in receptivity in Turkmenistan. Similarly, in Bhutan, electrification of homes and subsequent use of electric fans may have reduced transmission. Urbanization is another factor, in that it reduced the number and surface area of anopheline breeding habitats. Water bodies became dry or polluted in some provinces in the Philippines, leading to a decline in larval habitats, since the primary vectors require clear, clean, slow flowing water. For many case study countries, in particular in the Asia Pacific, primary vectors were forest dwelling. Increasing deforestation reduced vector-breeding habitats, such as in Sabah State of Malaysia, where the decline in forest habitat was believed to have reduced vector abundance of *Anopheles balabacensis*. Economic development in Mauritius in the 1950s and 1960s reduced malaria transmission, leading to the first malaria elimination campaign (1969) and helped to sustain lower transmission levels for the rest of the Century. Although receptivity may have declined in some countries, these transitions were also accompanied by increases in population movement or immigration into receptive areas, elevating the potential risk of transmission. This increased vulnerability due to risk of importation has affected Bhutan and Malaysia even while receptivity is declining.

While changes in economic or infrastructure development in some countries led to a decrease in receptivity, in some areas changes led instead to an increase in receptivity. Irrigation schemes increased levels of receptivity in several countries, such as in Turkey and Mauritius. Dam construction was thought to have increased receptivity in Sri Lanka, Turkmenistan and Bhutan. For example, in Sri Lanka, the 1987 epidemic was linked to a major dam construction project on the Mahaweli River, in the malaria-endemic eastern part of the country, which included forest clearing for rice cultivation. This change in land use resulted in an increase in receptivity, which increased risk of malaria for the one million settlers who moved there from non-endemic areas.

Cape Verde and the Philippines provide examples of the increase in receptivity due to human behaviour. In Mauritius, flat rooftops became popular after the 1960s but because of the pooling of water may have led to an increase in receptivity, as they provided good larval habitats for *Anopheles gambiae*. In the Philippines, the benefits of electrification in reducing transmission may have been offset in remote areas as more people stayed up later in the evening hours when vector exposure is greatest.

Some changes in development have accelerated malaria transmission or, in contrast, progress toward elimination. In either case, continuous measurement of receptivity will alert malaria programmes to changes in transmission dynamics. This measurement relies upon ongoing, robust entomological surveillance.

### Combining vector control strategies

Most programmes rely on a combination of interventions, which together are believed to have reduced vectorial capacities and receptivity of the risk areas.

IRS was a primary tool for most programmes, along with ITN/LLIN to increase coverage of vector control and some type of larval control. Some countries credited the combination of interventions with reducing incidence or receptivity in their countries. In Mauritius, IRS, space spray and larviciding were used in combination with surveillance in active foci; non-active foci receive all interventions except for IRS. The programme attributed success to the control of larval habitats above all other interventions. In the Philippines, the combination of IRS and LLINs was credited for the significant drop in cases since the 1990s. In Turkey, the impact of vector control methods was used as a justification for the setting of a national elimination goal, with the plan to use IRS, larvivorous fish and ITNs to reduce receptivity and achieve elimination.

## Discussion

In the elimination case study series, the scope of data collection was broad and not focused exclusively on vector control, which in some cases translated to a limitation in the comparability of results in the cross-case study analysis. Quality and coverage of vector control interventions was difficult to understand and to compare across case studies, limiting the lessons drawn across all the countries’ experiences. Furthermore, assessment of the impact of vector control interventions was either not available or not fully explored in any of the case studies, most attribution of impact was anecdotal. Moreover, there was no possibility to explore counterfactuals to compare interventions, or lack of, when analysing what may have helped or hindered the programme. However, even with these limitations, the case studies were used as the primary data source as they were comprehensive and extracted information from national malaria programme data, reports, and publications; WHO reports; malaria programme reviews; and WHO and other historical documents.

Some common themes and lessons have emerged. The cross-case study analysis showed that most countries, both eliminating and POR, employed a similar range of vector control tools in the latest period of elimination. IRS was a primary vector control tool throughout the case studies, as most countries have continued this intervention since the GMEP era, when it was proven effective at reducing receptivity. However, there were several examples of programmes that rapidly scaled down IRS without evidence of any strategic planning or stratification process. It is possible that reductions in IRS were linked with a foci- and case-based (focal) strategy, where cases declined and then IRS was phased out. However, this was not clearly described. Instead, the declines in IRS documented in the case studies appear to be more related to a reduction in funding, personnel, programme capacity, or due to ongoing operational constraints. Several countries slowed or halted IRS and subsequently had outbreaks or epidemics. More information is needed on how and when countries should consider decreasing or halting of their primary vector control interventions, and how to maintain capacity to respond to outbreaks. ‘Stopping’ or ‘slowing’ rules for vector control, or guidelines on when programmes should scale down IRS or LLIN distribution or halt them completely, would be helpful to countries pursuing and reaching elimination.

Other tools used by most countries included LLINs, in particular to provide prevention for hard-to-reach populations (e.g., in remote or unstable and insecure areas, or areas with a high number of mobile populations). In some case studies, LLIN use was directly linked with access to external funding, such as from the Global Fund.

Larval control and environmental management were implemented by many programmes, however, coverage and effectiveness were not well described in the case studies nor was the articulation of rationale supporting their use. There was a lack of evidence of effectiveness of these tools in reducing receptivity or malaria transmission by programmes, likely because it was challenging to measure or studies where it did not show impact were not reported. There was also scant research undertaken to measure effectiveness of environmental management schemes. Larval source management (not including larvivorous fish), in selected circumstances, has been found to contribute to a reduction in malaria incidence [23]. There was only “low quality” evidence reported in the Cochrane Review on larvivorous fish, where there was variable evidence of the effect of larvivorous fish on the density of larvae or reduction in breeding sites with immature vector breeding, and no studies measured the impact of larvivorous fish on malaria incidence [20]. Notwithstanding, if countries choose to rely upon larval control instead of IRS and/or LLIN implementation as they approach elimination, more country-level and setting-specific evidence, based on rigorous evaluation, is still required for more consolidated conclusions [24].

The objective of implementing IVM was not well articulated by the malaria programmes, and the meaning of this strategy varied across programmes. While it means a combination of five components, most programmes assumed that intervention combination was the main IVM strategy.

Countries in the case study series that have successfully eliminated malaria and are now in the POR phase had similar approaches. All POR countries used IRS and larval control as primary vector control measures. Two of the three countries that successfully reached elimination combined IRS with other interventions with the intention of reducing receptivity. POR countries had a more detailed description of the entomological surveillance activities undertaken, which appeared to be consistently implemented over time.

As entomological surveillance data should be the basis for all response interventions and programme strategies, consistent and high-quality data are needed [25]. Entomological surveillance was prioritized by some programmes, in particular in countries that are either close to or have achieved elimination. However, the response component of this surveillance, which could be used for outbreak forecasting, stratification leading to targeting of interventions, and longer term malaria programme strategy, was either not a programme intervention or was poorly articulated in the case studies. Information on insecticide resistance monitoring was scarce, with only a few reports of insecticide resistance and the programme response. There were limited data on how entomological surveillance was conducted or the workforce needs, and no description of collaboration with reference or other research laboratories or training institutions. Linkage between the entomological and epidemiological data was not described, except in Malaysia, where one database combines both types of data. It is likely that most programmes were not taking advantage of these data to inform their intervention responses, coverage, timing or tools.

The choice of vector control tools in the case studies was not strongly linked to evidence. Although biologically plausible, the empirical evidence base on the effectiveness and cost effectiveness of vector control tools implemented, such as larviciding, environmental management and space spraying or fogging, remains weak. WHO does not recommend space spray [26]. Given that these interventions are implemented as part of integrated vector control strategy, it is difficult to conduct trials. However, countries embarking on introducing these interventions should consider incorporating rigorous operational research to gather evidence on the effectiveness and cost-effectiveness of these interventions.

Choice of vector control tools was not described as a response to the receptivity profile of the country. In fact the factors behind intervention choice were generally opaque across the case studies, leading to the assumption that there must be other background factors at play that are not articulated in the case studies. Global guidance, such as the 1993 WHO Global Malaria Strategy, likely informed some of these choices. Intervention cost, funding availability, and programme capacity required for distribution and operation of interventions were all likely factors at play, as well as cultural and historical factors.

## Conclusions

Scaling up or down of vector control, in particular IRS, was not linked clearly with changes in stratification, epidemiology or operational information. In most cases declines appeared to be decided based on funding constraints rather than strategy. The scaling down of IRS contributed towards malaria resurgence in several countries, wiping out years of effort and progress. Countries must be able to make a case to policy and decision makers for continued investments in vector control in order to ‘go the last mile’ and attain and sustain elimination. Programmes must be able to link together quality entomological surveillance data, evidence-based real-time vector control response strategies, evidence on impact of vector control, and comparable coverage and quality indicators to make this case. The linkage between epidemiological surveillance data and vector control as part of the surveillance and response intervention is critical as countries move towards elimination and seek to prevent resurgence. This entails a much closer link between the eco-systemic and public health approaches in malaria control and elimination. An evidence-based stratification system, using risk and receptivity maps, would help programmes make the case for maintaining coverage of risk areas with expensive and time-consuming vector control interventions [27].

The GTS provides a strategy of the action needed to accelerate progress towards elimination and AIM when placed in the context of a given country, and provides the framework for policy and advocacy. The international malaria community can take forward these strategies and play an important role in filling in the gaps that are outlined in this analysis of country experience. More work needs to be done to fill gaps in programme guidance, providing clarity on the best methods for choosing and targeting vector control interventions, and then supporting countries in the next steps, which are measuring cost, cost-effectiveness and cost-benefit of vector surveillance and control interventions.

## Abbreviations

AIM: Action and Investment to Defeat Malaria
GMEP: Global Malaria Eradication Programme
GPS: global positioning system
GTS: Global Technical Strategy for Malaria, 2016-2030
IRS: indoor residual spraying
ITN: insecticide-treated net
IVM: integrated vector management
LLIN: long-lasting insecticide-treated bed nets
POR: prevention of re-introduction
RBM: Roll Back Malaria
WHO: World Health Organization
WHOSIS: WHO Statistical Information System
API: annual parasite index
UCSF: University of California, San Francisco
